# KSHV (HHV8) vaccine: promises and potential pitfalls for a new anti-cancer vaccine

**DOI:** 10.1038/s41541-022-00535-4

**Published:** 2022-09-20

**Authors:** Corey Casper, Lawrence Corey, Jeffrey I. Cohen, Blossom Damania, Anne A. Gershon, David C. Kaslow, Laurie T. Krug, Jeffrey Martin, Sam M. Mbulaiteye, Edward S. Mocarski, Patrick S. Moore, Javier Gordon Ogembo, Warren Phipps, Denise Whitby, Charles Wood

**Affiliations:** 1 Infectious Disease Research Institute, 1616 Eastlake Ave. East, Suite 400, Seattle, WA 98102 USA; 2 Vaccine and Infectious Disease Division, Fred Hutchinson Cancer Research Center, 1100 Fairview Ave N, Seattle, WA 98109 USA; 3 Laboratory of Infectious Diseases, National Institutes of Health, Bldg. 50, Room 6134, 50 South Drive, MSC8007, Bethesda, MD 20892-8007 USA; 4 Lineberger Comprehensive Cancer Center & Department of Microbiology and Immunology, The University of North Carolina at Chapel Hill, Chapel Hill, NC 27599 US; 5 Department of Pediatrics, Vagelos College of Physicians & Surgeons, Columbia University, 630 West 168th Street, New York, NY10032 US; 6 PATH Essential Medicines, PATH, 2201 Westlake Avenue, Suite 200, Seattle, WA USA; 7 HIV and AIDS Malignancy Branch, National Cancer Institute, Bethesda, MD 20892 USA; 8 Department of Epidemiology and Biostatistics, University of California, San Francisco, CA USA; 9 Division of Cancer Epidemiology & Genetics, National Cancer Institute, NIH, HHS, 9609 Medical Center Dr, Rm. 6E118 MSC 3330, Bethesda, MD 20892 USA; 10 Emory Vaccine Center, Emory University, Atlanta, GA 30322 USA; 11 Cancer Virology Program, Hillman Cancer Center, University of Pittsburgh, Pittsburgh, PA 15213 USA; 12 Department of Immuno-Oncology, Beckman Research Institute of City of Hope, Duarte, CA 91010 USA; 13 Vaccine and Infectious Disease Division, Fred Hutchinson Cancer Center; Department of Medicine, Division of Allergy and Infectious Diseases, University of Washington, Seattle, WA USA; 14 AIDS and Cancer Virus Program, Frederick National Laboratory for Cancer Research, Frederick, MD USA; 15 Department of Interdisciplinary Oncology, Louisiana State University Health Sciences Center, New Orleans, LA 70112 USA

**Keywords:** Tumour virus infections, Epidemiology

## Abstract

Seven viruses cause at least 15% of the total cancer burden. Viral cancers have been described as the “low-hanging fruit” that can be potentially prevented or treated by new vaccines that would alter the course of global human cancer. Kaposi sarcoma herpesvirus (KSHV or HHV8) is the sole cause of Kaposi sarcoma, which primarily afflicts resource-poor and socially marginalized populations. This review summarizes a recent NIH-sponsored workshop’s findings on the epidemiology and biology of KSHV as an overlooked but potentially vaccine-preventable infection. The unique epidemiology of this virus provides opportunities to prevent its cancers if an effective, inexpensive, and well-tolerated vaccine can be developed and delivered.

## Introduction

Follow-up of the Cancer Moonshot’s success requires practical, new, and effective anticancer preventions and therapeutics^1^. It is not widely recognized but approximately one-sixth of all human cancer cases result from only seven viruses, causing well over a million deaths each year^2^. Preventive vaccines developed decades ago against human papillomaviruses (HPV)^3^ and hepatitis B virus (HBV)^4^ reveal the effectiveness of simple vaccinations to reduce the cancer burden. While these two vaccines engender a blockbuster global public health success story, surprisingly little headway has been made by the general cancer research community to extend these results to prevent—or treat—other viral cancers.

Rapid and successful development of SARS-CoV-2 vaccines compels revisiting this issue. In October 2021, the National Cancer Institute’s Office of HIV and AIDS-related Malignancies organized an open meeting of over 100 experts and stakeholders to discuss the feasibility of vaccines to control Kaposi sarcoma herpesvirus (KSHV), also known as human herpesvirus 8 (HHV8). This was the first meeting of its kind to explore the worldwide KSHV cancer burden and to raise the issues on whether and how KSHV vaccines should become a research and public health priority. Currently, there are no well-developed KSHV vaccine candidates and little funding for KSHV vaccination research.

This meeting addressed critical questions on KSHV cancers and vaccines: What is the cancer burden from KSHV? If an effective vaccine is developed, who should be vaccinated? Would an effective vaccine be preventive by providing sterilizing immunity or therapeutic by controlling the virus-cancer after infection? Would a successful vaccine rely on neutralizing antibodies to KSHV antigens, cell-mediated immunity, or combinations of both? Finally, if an obvious and effective vaccine is developed, what are the barriers to real-world implementation of KSHV vaccination and how can they be overcome?

The participant consensus was surprisingly optimistic: KSHV is biologically very different from its more ubiquitous herpesviral cousins in that it is not common in some populations, consistent with limited viral transmissibility and high susceptibility to immune surveillance that could be bolstered by vaccination. On an evolutionary timescale, KSHV is likely to have been lost from many populations in the world, raising the possibility that artificially stimulated immunity can readily increase this trend^5^. Further, the DNA viral genome is highly conserved—particularly in its structural genes—suggesting a single effective vaccine will prevent all variants in different world regions.

Though poorly recognized outside of the KSHV research community, there is a clear public health need for KSHV prevention that extends beyond the realm of HIV and AIDS^6^. KS under-reporting, particularly in cancer registries, is a major barrier to assessing this need^7^, particularly for low- and middle-income countries. Over 90% of the adults in some rural areas of East Africa are KSHV-infected^8^. This region has high rates of KSHV cancers in both HIV-positive and negative populations. Belts of high KSHV cancer endemicity extend throughout Africa up into Mediterranean and Western Asian populations and into populations of South America^6,9–11^. In high-income countries of Europe and North America, men who have sex with men (MSM) and organ donor recipients remain at high risk for KSHV-related cancers, and hence targeted vaccination programs could be envisioned^12^.

**Fig. 1:**
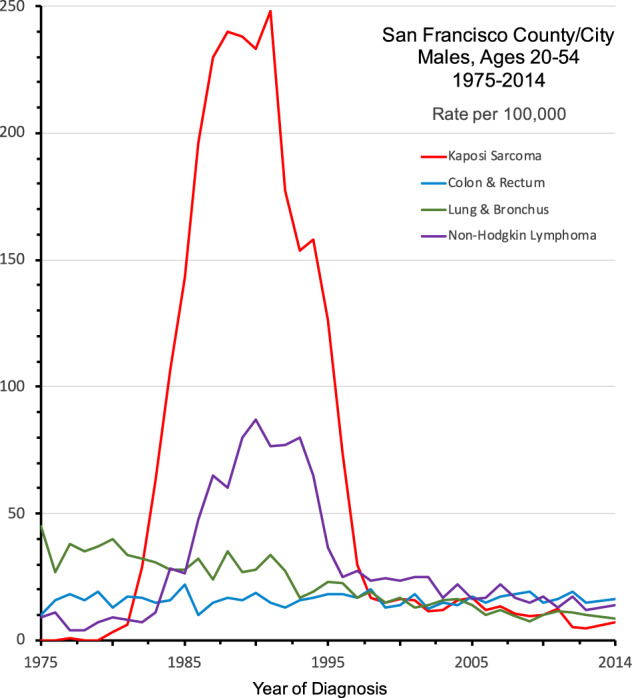
The Kaposi sarcoma epidemic. U.S. National Cancer Institute’s Surveillance, Epidemiology and End-Results (SEER) data for San Franciscan men shows the emergence and magnitude of this epidemic cancer caused by transmissible KSHV infection during the AIDS epidemic (Howlader, N., Noone, A.M., Krapcho, M., Miller, D., Bishop, K., Kosary, C.L., Yu, M., Ruhl, J., Tatalovich, Z., Mariotto, A., Lewis, D.R., Chen, H.S., Feuer, E.J., Cronin, K.A. (eds). SEER Cancer Statistics Review, 1975–2014, National Cancer Institute, Bethesda, MD, https://seer.cancer.gov/csr/1975_2014/).

As revealed by the AIDS epidemic (Fig. 1), KSHV infection is, first and foremost, a communicable disease, which can silently increase in any population due to changes in behavior norms or other risk factors. This has similarly occurred as seen by the current epidemic of HPV+ head and neck cancers in men related to changes in oral sexual practices^13^. Similar social changes could set the stage for the emergence of KSHV in populations in which it was previously rare, as already occurred among gay and bisexual men in the US and elsewhere in the 1970s–80s. Since primary KSHV infection is generally asymptomatic and ongoing KSHV surveillance is minimal, research and development on effective KSHV vaccines are prudent to address future KSHV transmission and pandemics caused by changes in the virus, host, or society.

## Kaposi sarcoma and related cancers

Kaposi sarcoma (KS) was first described in 1872 by Moriz Kaposi as an aggressively malignant skin tumor^14^. Later studies revealed that KS is an endothelial cell cancer that is often indolent^15^. For some time, it remained a clinical curiosity, mainly manifested as a relatively rare cancer among the Mediterranean, particularly Ashkenazi Jewish, populations^15^. It was subsequently found to be common among sub-Saharan African (SSA) populations, particularly in the Central and Southeastern belt of African countries where it was the second to third most common malignancy in the general pre-AIDS populations^9,16^. KS cases in the US markedly increased in the 1960s–70s with the development of organ transplant-related therapies, focusing attention on the immune system to control this cancer^17^.

The onset of the AIDS pandemic in 1981 was heralded by the epidemic of KS, mainly among previously healthy MSM^18^. The major AIDS-related cancer burden, however, has been highest among African populations where, for example, KS rose 10-fold, from 5% to 49% of all adult Ugandan male cancer patients, with the emergence of AIDS^16^. Despite effective anti-retroviral therapies, KS remains leading cancer among HIV/AIDS patients worldwide^19^. KSHV was discovered in 1994 as the cause of all forms of KS, including various manifestations of HIV-related and unrelated KS, and lifelong infection with KSHV clearly predisposes to the development of KS^20^. In addition to KS, other malignancies associated with KSHV include primary effusion lymphoma (PEL) and one of the forms of multi-centric Castleman disease (MCD) occurring primarily among persons with HIV/AIDS^19^.

## The global distribution of KSHV infection

The epidemiology of KSHV infection has been recently summarized by Cesarman et al.^21^. Even with imperfect antibody testing^22^, the worldwide distribution of KSHV infection can be clearly delineated^21^. SSA is by far the region with the highest KSHV seroprevalence, in both children and adults, where the virus is mainly transmitted through non-sexual, but as yet undefined, behaviors and practices most probably related to saliva exposure^23,24^. Estimates are that ~20–80% of the general SSA adult population are persistently KSHV-infected, with the variability often reflecting either geographic or assay performance differences. Mirroring historically high KS rates in SSA countries prior to AIDS, serum banks collected in the 1970s from East African populations have KSHV antibody positivity of >80% among adults^25^. Childhood KSHV infection is acquired through non-sexual transmission and is common in SSA countries^26^ but is uncommon or rare in most other portions of the world. Pediatric KS, which is also frequent SSA countries^27^, can have a fulminant lymphadenopathic presentation that is rapidly fatal in both HIV-infected and HIV-uninfected patients^28,29^. Thus, prevention of pediatric KSHV infection will have important public health consequences, particularly in highly endemic areas of SSA.

In contrast, adult seroprevalence in the populations of northern Europe and North America^30^ is generally low (usually <5%) with increased prevalence in some Mediterranean populations, (e.g. Southern Italy) with up to ~20–40% of persons commonly reported. In these otherwise low seroprevalence areas, MSM (who are often excluded from some blood donor serosurveys leading to an underestimation bias) often harbor the highest prevalence of KSHV infection with rates rivaling those seen in SSA. In the US, South America, Europe, and Australia, 30–65% of HIV-infected MSM and 20–30% of HIV-uninfected MSM are KSHV antibody-positive^31–33^.

**Fig. 2:**
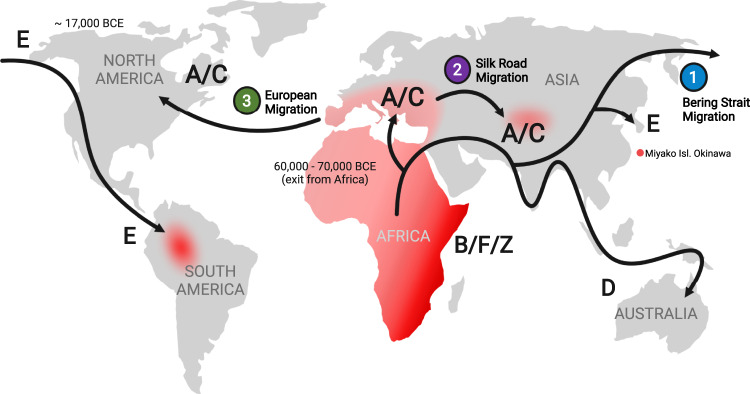
Global patterns of KSHV spread with KSHV endemic and hyperendemic areas (red). Molecular epidemiology studies indicate that KSHV evolved with humans and spread primarily through human migrations. KSHV D and E strains may represent modern remnants of an ancestral KSHV strain migrating with early humans that was subsequently lost from most Asian and European populations after settlement, leaving endemic and hyperendemic geographic pockets in Okinawan islands and in South America. Additional viral strains (e.g., A, B, C, and Z) appear to have re-emerged in Africa and the spread of some strains may represent secondary out-migrations to Mediterranean, Western Chinese, and North American populations within historical periods. Unlike other human herpesviruses, KSHV is not ubiquitous and current global patterns are consistent with the potential for human immune clearance of this virus.

Similar to Europe and North America, KSHV prevalence is low in most populations of South and East Asia, consistent with the virus likely being lost after human outmigration through this region (Fig. 2), and higher prevalences being present among MSM. Isolated high KSHV and KS rates among indigenous Okinawan^34^ and South American^35^ populations may reflect ancient original co-migration of the virus with human populations^36^. High KSHV seroprevalence is seen in some Western Asian populations^10,37^ that may reflect a historical reintroduction of KSHV from Mediterranean trading routes (the Silk Road hypothesis) to Chinese populations^5^. However, serologic studies in this region are uneven, and if vaccine development moves forward, it will be essential to re-survey representative populations using up-to-date and reliable assays to determine if the initially-described patterns of KSHV distribution persist.

The modes of KSHV transmission vary in high versus low KSHV endemicity areas. In SSA, transmission often occurs in pre-pubescent children prior to the age of sexual debut^38^. This pattern of infection suggests that horizontal non-sexual spread predominates in this setting, consistent with direct parent-to-child or child-to-child transmission via saliva as occurs with other herpesviruses. Outside of SSA, most transmission research has focused on MSM, in whom KSHV antibody-positivity rapidly increases after sexual debut and is highly correlated with the number of intimate male partners^21^. Despite attempts to delineate specific modes of person-to-person spread, the specific mechanisms of spread are unclear but are likely to involve sexual or non-sexual saliva transmission. Methodologically, the inherent difficulties associated with distinguishing the roles of tightly correlated behavioral acts in MSM (e.g., use of saliva as a sexual lubricant, oral–anal contact, penile–oral intercourse) make this research challenging.

Although epidemiologic studies have been unable to define specific routes of transmission, the clinical virology of KSHV has narrowed the possibilities and has implications for vaccine efficacy. Among bodily fluids readily passed between humans, saliva by far harbors KSHV most frequently^39–50^. KSHV DNA has occasionally been detected in genital fluids and breast milk, but at a lower frequency and lower quantities relative to saliva^40,43^. These findings favor contact with saliva as the medium likely to favor KSHV transmission and suggest the promotion of vaccines that generate mucosal immunity. The acts that spread saliva, however, are diverse including those heretofore under-appreciated (e.g., use of saliva as an anal lubricant among MSM and use of saliva to soothe insect bites among African children). Abrogation of KSHV oral shedding among KSHV-infected persons might be another objective for vaccines in which the measurement of viral DNA in saliva could be used as a vaccine efficacy end-point.

Because shedding in the saliva is intermittent^45,47^, cross-sectional assessments may underestimate transmission risk and are likely to be insensitive as markers of vaccine efficacy if sterilizing immunity is the goal. The prevalence of oral shedding among seropositive persons varies from 3% to 100%, based on studies conducted among KSHV-infected women^45^. A study of seropositive participants from SSA, US, and Peru found that about 40% were frequent or persistent oral shedders for 20 or more days in a 30-day period of observation^47^. These data point to the frequency and quantity of KSHV shedding in saliva as a potential target for efforts to reduce KSHV transmission.

A few factors have been associated with the frequency or quantity of KSHV detection, such as male sex^43,44,48,50^, younger age, particularly in SSA^41,44,50^, and higher lytic KSHV antibodies, e.g. anti-K8.1^41,42,48–50^. In SSA, parasitic infections (malaria, helminthiasis) are associated with increased KSHV detection in blood, but their relationship with shedding in saliva has yet to be determined^48,50^. HIV infection is a risk factor for KSHV DNA detection in blood and KS, but the relationship with KSHV shedding in the saliva is unclear^40,43,49^. It will be essential to study representative populations to better characterize factors that influence shedding to inform decisions about the value of KSHV shedding as a marker of vaccine efficacy.

## The place for a KSHV vaccine

High KS rates in some HIV-uninfected populations, particularly in SSA and western China^9,12,51,52^ have often been ignored as a public health burden. Also, while access to antiretroviral therapy (ART) has proved a potent intervention to prevent AIDS-KS in resource-rich countries, ART availability in low- and middle-income countries (LMIC) has not been uniform. Furthermore, KS incidence has fallen less dramatically in SSA after ART rollout compared with reductions seen in the Global North^53^. Despite the effectiveness of ART in preventing AIDS-KS, it does not eliminate KSHV latency so the resurgence of KSHV-related cancers in aging HIV/AIDS patients remains a significant concern in both high-income and LMIC countries.

For SSA populations with high childhood KSHV transmission, the introduction of a childhood sterilizing preventive vaccine could interrupt childhood KSHV transmission and prevent KS. This strategy could be integrated broadly into the Expanded Program for Immunization. Outside of SSA, vaccination might be geared towards high-risk populations based on local geographic regions (e.g., Southern Italy) or risk groups (e.g., MSM, prospective transplant recipients) for low-prevalence countries, instead of universal vaccination. For a solely preventive vaccine to be effective, it must be given prior to virus exposure. Given the evidence supporting KSHV as a sexually transmitted infection in low-prevalence countries, an approach similar to HPV vaccination might be effective, with administration prior to the onset of sexual debut. In contrast, a therapeutic KSHV vaccine to prevent cancer development regardless of prior infection may have public health importance to protect high-risk individuals (MSM or potential organ recipients) in low-prevalence countries. Whether women should be considered for vaccination in low prevalence countries depends on changes in social behaviors that might lead to an unexpectedly increased risk for KSHV infection and/or KSHV-related malignancies.

Although the lack of precise knowledge about how KSHV is spread is a limitation, KSHV bears many epidemiological similarities to HBV that has similar limitations, and for which a vaccine was successfully developed and implemented^54^. Notably, the universal administration of HBV vaccination during infancy in regions endemic to HBV has nearly eliminated hepatocellular carcinoma in vaccinated infants and young adults^55^. An effective vaccine would provide a long-term preventive solution to the risk of KSHV malignancies that might emerge during future social changes, such as the expected increase in immune suppression prevalence due to increasing access to solid organ transplantation in SSA.

## Antigen targets for a KSHV vaccine

**Fig. 3:**
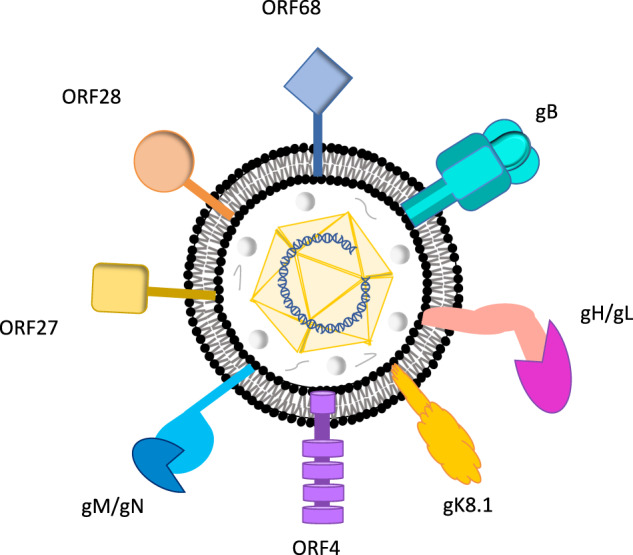
Schematic diagram of major Kaposi sarcoma herpesvirus membrane structural glycoproteins. Individual viral components are indicated. The viral genome is a linear double-stranded DNA enclosed in an icosahedral capsid. This nucleocapsid is surrounded by the tegument. The tegument is a dense amorphous proteinaceous structure. The viral envelope is a lipid bilayer membrane of host origin that contains ten different KSHV glycoproteins. Conserved glycoproteins that are potential preventive vaccine antigens include gB, gH, gL, gM, and gN, and accessory glycoproteins gK8.1, ORF4, ORF27, ORF28, and ORF68.

To design an effective vaccine against KSHV, the identification of viral antigens that are detected by particular arms of the host immune system is critical (Fig. 3, Table 1)^56^. KSHV, and related herpesviruses, differ from many other viral infections in having a life-long, persistent (latent) infection. An ideal KSHV vaccine may need to elicit both antibody responses to induce sterilizing immunity to prevent primary infection as well as T cell responses for protection against cancers in latently infected individuals. The use of the whole virus as a candidate antigen is an attractive option but also has potential limitations. A live virus vaccine modeled on the successful herpesvirus Oka strain varicella-zoster virus vaccine would require extensive engineering of putative KSHV oncogenes to ensure safety concerns are met for this cancer virus. Inactivated whole virus, in contrast, provides a simple and inexpensive alternative approach but it is unknown if this can be used to artificially induce effective protective immunity.

**Table 1:** KSHV antigens that might become the basis for effective KSHV vaccines.

	Common name	Gene	Function	Reference
Structural proteins	gB	ORF8	Glycoprotein, cell fusion	^125^
	gH	ORF22	Glycoprotein, binds ephrin receptors	^75^
	gL	ORF47	Glycoprotein, binds ephrin receptors	^75^
	K8.1	ORFK8.1	Glycoprotein, binds heparan sulfate	^126^
	KSHV complement-binding protein (KCP)	ORF4	Glycoprotein, binds heparan sulfate and complement	^127^
	gM	ORF39	Glycoprotein, cell fusion	^77^
	gN	ORF53	Glycoprotein, cell fusion	^77^
	ORF27	ORF27	Glycoprotein	^128^
	ORF28	ORF28	Glycoprotein	^129^
	ORF68	ORF68	Glycoprotein	^130^
Non-structural proteins	Latency-associated nuclear antigen 1 (LANA1)	ORF73	Latent genome maintenance	^131^
	K1	ORFK1	Signaling glycoprotein	^132^
	Viral Interleukin 6 (vlL6)	vlL6	Homolog to human IL6	^133^
	Viral G protein-coupled receptor (vGPCR)	ORF74	Signaling GPCR	^134^

### KSHV immune responses during natural infection

KSHV infection among asymptomatic persons is generally determined by seropositivity to two viral antigens, K8.1 protein and latency-associated nuclear antigen, LANA^57^. Using assays to these antigens, individuals with the clinically apparent disease have higher antibody levels compared to those without the disease who nevertheless were previously exposed and carry the latent virus. Surveys using recombinant KSHV proteins reveal that antibody responses to other KSHV proteins are highly heterogenous but can help discriminate between KSHV-infected persons and uninfected donors^25,58^.

To be successful, a KSHV vaccine will need to generate more potent and specific neutralizing antibody and/or cell-mediated immune (CMI) responses than occurs during natural infection^59^. Measuring cellular immune responses to natural KSHV has proven challenging. Only a handful of studies have examined cellular immune responses to KSHV infection and in general show a lack of cell-mediated immunodominance^60^. Further, during natural virus infection, KSHV encodes multiple mechanisms to subvert an effective immune response that otherwise would allow clearance of the viral genome, particularly by skewing T cell responses through the expression of multiple cytokines/chemokines and through cis-protein coding mechanisms^61,62^. The nonstructural (NS) protein LANA is required for viral persistence and KS patients can have anti-LANA antibody titers >1:100,000^63^ but this antibody response is ineffective against this intracellular antigen. Evidence also indicates that superinfection of previously exposed individuals is possible, suggesting that prior natural infections to the virus do not generate sufficient sterilizing immunity to prevent secondary infections^64^. Ideally, a KSHV vaccine should provoke sufficient memory B and T cell responses to continue to give protection even if the vaccinee later develops HIV CD4+ lymphopenia.

### Preventive vaccines: KSHV glycoproteins

A preventive KSHV vaccine is likely to be based on neutralizing antibody responses to viral glycoproteins required for cell entry so that systemic infection and latency cannot be established (Fig. 3)^65^. CMI responses to structural proteins may also contribute to sterilizing immunity but, in contrast to antibody responses, have not been extensively studied. There is a practical advantage to assessing preventive KSHV vaccines since an efficacy end-point can be determined using seroconversion to non-structural latency antigens (e.g., LANA) that are not present in the vaccine. With extremely high KSHV seroconversion rates occurring in some populations, such as East African children^26^, this study design would be able to use smaller cohorts followed over a shorter period of time than cancer prevention trials, and would both simplify and improve the cost-effectiveness of the vaccine trials. One key lesson for preventing KSHV infection comes from the closely related murine gammaherpesvirus 68 (MHV68), in which a single infectious particle is predicted to be sufficient for establishing chronic infection in mice^66^. The MHV68 model also reveals that the mode of antigen administration and antibody-independent immunity may be important for a preventive KSHV vaccine. In one study, initial nasopharyngeal administration of MHV68 prevented systemic super-infection during the secondary nasopharyngeal challenge and this was achieved through local nasopharyngeal CMI responses rather than elevated antibody responses^67^.

KSHVcauses a systemic infection and displays a broad tropism, including epithelial cells, endothelial cells, fibroblasts, keratinocytes, lymphocytes, and monocytes, in which multiple viral entry receptors have been identified^68–70^. KSHV glycoproteins K8.1, ORF8 (gB), ORF22 (gH), ORF 46 (gL), ORF39 (gM), and ORF53 (gN) are virion-associated and are prime candidates for KSHV attachment, fusion, and entry functions^68,71–76^. The gN/gM complex appears to play a role in egress rather than viral entry^77^. In the current model of KSHV entry, the first viral contact is mediated by envelope-associated K8.1 and gB binding to heparan sulfate (HS) on the host-cell surface^68,71,78^. Although this binding is reversible and not critical for entry, it localizes the virus on the cell surface, providing the initial direct contact between viral glycoproteins and specific cellular entry receptors^79,80^. Upon binding, conformational changes allow the gH/gL complex to access specific host-cell receptors, including integrins and ephrin receptors A2^73^, EphA4^81^, and EphA7^82^. This is followed by fusion of the viral envelope via gB with endosomal membranes, the release of the viral capsid in the cytosol, trafficking of the capsid to the nuclear membrane, and delivery of the KSHV genome into the nucleus^83^. Additionally reported cell receptors for KSHV include cystine/glutamate antiporter (xCT) and dendritic cell-specific intercellular adhesion molecule-3-grabbing non-integrin (DC-SIGN), which may allow entry into various cell compartments^84^. The complexity of these processes suggests a vaccine directed at eliciting antibodies to multiple epitopes (dominant and subdominant) should be considered.

KSHV K8.1, gB, and gH/gL were recently tested as components of a virus-like particle-based candidate vaccine^85–87^. This resulted in KSHV-neutralizing antibodies in immunized mice and rabbits that prevented in vitro infection of epithelial, endothelial fibroblast, and B cells. This suggests that these glycoproteins might hold an important role in viral entry, but only the role of gH/gL has been thoroughly studied in multiple cell types^85,88,89^. In these studies, gH/gL was shown to be indispensable for epithelial, endothelial, and fibroblast cells^85,88,89^, but whether this holds true in vivo in the oral cavity is unknown. KSHV is present in the saliva and salivary shedding is widely accepted as the primary conduit of KSHV transmission^21,23,24,90–92^. Using a similar approach, K8.1 was shown to be dispensable for entry into epithelial cells in vitro but not into B cells^93,94^. Research on conserved glycoprotein-based vaccines for other herpesviruses^95^ may provide helpful guidance for the development of a KSHV vaccine.

### Other KSHV antigen targets

Little systematic research is available on non-structural protein targets for potential KSHV vaccines. Unlike KSHV structural proteins, which are highly conserved and even cross-reactive to other herpesvirus entry proteins, KSHV encodes an array of nonstructural proteins that are unique to the virus. The LANA1 genome maintenance protein is constitutively expressed in all KSHV-infected cells and may be a good candidate for CMI-based vaccines via mRNA delivery if the antigen is modified to eliminate its CMI-escape motif^62^. As an intracellular nuclear antigen, it is unlikely to be targeted by humoral immunity. Evidence suggests that CMI during natural infection against LANA and other antigens is heterogenous and may not limit virus replication, so CMI-based vaccine targets may have to be individually tested to determine if the enhanced immunity from vaccination provides protection^59^. Several KSHV nonstructural proteins potentially critical for viral persistence are expressed on the infected cell-extracellular surface or are secreted and that could, in theory, be neutralizing targets for vaccine-induced antibodies. These proteins include the viral K1 protein^96^, vIL-6^97^, viral G protein-coupled receptor^98^, and viral chemokines^99^, as well as other potential antigens. The heterogeneity of both antibody and cellular immune responses to KSHV suggests that until a singular viral protein has been validated as the dominant target, vaccine candidates with as many viral antigens as possible may be preferable and platforms incorporating inactivated viruses or live-attenuated viral vectors, virus-like particles expressing multiple viral antigens, or multivalent RNA vaccines may be needed. Replicating vaccine viruses engineered to remove latency and antiviral effectors represents another promising strategy as they present a wide breadth of antigens and provide durable protection^100^.

Although an ideal vaccine would induce sterilizing immunity, this may prove to be challenging and it may not be necessary to reduce KSHV-associated morbidity and mortality. A program to define human monoclonal antibodies that block infection in in vitro cell culture and for animal models^101^ might be a useful prelude to help define an ideal preventive vaccine antigen. The success of adoptive immunotherapy trials against EBV-related malignancies^102^ suggests that a boost in the breadth and magnitude of adaptive T cell responses may confer protection against KSHV-related disease. Whether this could possibly clear the latent viral infection and prevent KSHV reinfection in healthy persons remains to be explored. Vaccines that elicit oral IgA are also attractive in interrupting salivary KSHV transmission. Additional investigations on the immune correlates of KSHV infection and KSHV-related disease are needed to assess whether these vaccine strategies can be beneficial.

## KSHV vaccines for low- and middle-income countries (LMIC)

The development of an effective vaccine candidate that can prevent KSHV infection and onward transmission or prevent secondary tumorigenesis after infection is a tractable scientific problem. But even if an ideal vaccine candidate is developed, implementing sustainable and economically viable public health programs devoted to KSHV prevention will face major hurdles.

KS is a malignancy with complex epidemiology, and an overwhelming number of cases occur in low-income countries^11^. Consequently, a vaccine against KSHV will need to not just be well-tolerated and immunogenic, but also must be inexpensive to manufacture and simple to distribute/deliver. In regions of the world where KSHV is endemic, the infection begins in childhood but continues through adulthood^103^. The ideal target product profile of a vaccine against KSHV would therefore include the ability to safely deliver a vaccine to infants that is durable, thermostable, and potentially needleless to enhance acceptance in this age group.

The only gamma-herpesvirus vaccine to advance to Phase 2 testing in humans is an Epstein Barr Virus (EBV) envelope glycoprotein with a potent toll-like receptor 4 adjuvant formulation^104^. Subunit protein vaccines with modern adjuvant formulations have been shown to be safe in children, highly immunogenic, thermostable, and durable^105,106^. RNA vaccines have also been designed against herpesviruses including cytomegalovirus (CMV)^107^ and EBV^108^, and may eventually be a promising vaccine platform for KSHV in the future should costs of manufacturing decrease. As with SARS-CoV-2 mRNA vaccines, thermostability for a KSHV mRNA vaccine might be a critical barrier in some LMIC. Taken together with promising recent advances in the thermostability of adjuvanted protein^105^ and RNA vaccines^109^, preventative KSHV vaccines should be encouraged for development on these platforms. New technologies such as mRNA vaccines, the use of multivalent virus-like particles, self-assembling proteins, and novel viral vectors capable of encoding multiple antigens all offer potential platforms for delivering antigens once protective immune responses are known.

## Target product profiles for KSHV vaccines

Even in the early stages of KSHV vaccine research, vaccine target product profiles (TPP) can guide the choices for antigens and methods used for vaccine manufacture and delivery (Box 1). The development of a TPP will be critical for establishing vaccine efficacy in clinical trials. KSHV might be completely controlled, for example, by prior vaccination in persons who develop acquired immunodeficiency so long as immunity is induced prior to the compromised immune function^110,111^. Prior vaccination could be practical for prophylaxis of infants or children in high incidence areas of the world such as SSA, Western Asia, and Eastern Mediterranean countries if immunity is lasting. Varicella zoster virus is the only human herpesvirus for which vaccines^112^ have been licensed both for acute infection (varicella) and for reactivation (zoster)^112,113^, although Epstein-Barr virus has also been targeted with promising efficacy in virus-naïve populations when a subunit gp350 in the adjuvant vaccine was evaluated^104^.

In describing the TPP most likely to succeed, it is important to recognize several limitations of KSHV vaccine research and development:

- There is no ready or predictive animal model for KSHV-related cancers (although animal models are valuable to assess KSHV infection^101,114,115^). Preclinical studies may be advanced using related viruses such as the murine MHV68^116^.
- Vaccine design should be geared toward safety, avoiding predicted severe adverse events. Since KSHV is a tumor virus, disabling viral oncoproteins are likely to be required for subunit DNA-based or live virus vaccines.
- Determining efficacy against tumorigenesis might require multi-year evaluations or prohibitively large clinical trials. Depending on the vaccine, either defining smaller high-risk populations likely to develop malignancy (e.g., newly diagnosed AIDS patients without KS in a high KS prevalence area) or surrogate measures that can reasonably predict efficacy, may overcome this limitation. Surrogate measures, for example, could include protection against infection (measured by antibodies against KSHV antigens not included in the vaccine), high-level immune responses, or prevention of oral shedding.
- Government and non-governmental organization support for the development and deployment of a vaccine are critical since this viral disease mainly targets socially and economically marginalized populations.

Box 1 Possible KSHV Vaccine Target Product Profile Criteria Vaccine target product profiles (TPP) are used to predict practical problems and issues in vaccine development, efficacy testing, manufacture and ultimate use of vaccine candidates.

*Goal*: Prevent primary KSHV infection? Prevent progression to disease in previously KSHV-infected population?

*Target populations*: Children and adults in high incidence, low-income areas? High-risk adults in low-incidence, medium- or high-income areas (e.g., Gay and bisexual men, organ transplant recipients)? HIV+ vs. HIV− populations?

*Main criterion for success*: Vaccine efficacy to prevent KSHV infection? Vaccine efficacy to prevent KS disease? *Secondary criteria*: Decrease other KSHV-associated illnesses?

*Formulation*: Subunit/adjuvant, inactivated virus, attenuated live virus, DNA- or RNA-based?

*Immune mechanism*: Induction of sterilizing antibody immunity? Induction of CD4+ and CD8+ T cell immunity?

*Dosing and administration*: One or two-dose intramuscular injection? Oral delivery for the mucosal-based vaccine?

*Safety*: Well-tolerated without serious adverse events

*Storage*: Lyophilized antigen? Cold-chain requirement?

*Cost*: Including manufacture, distribution, and delivery of the vaccine.

*Value proposition*: Reduce the lifelong impact of KSHV-associated diseases, cost–benefit analyses?

## Vaccine development and implementation for low- and middle-income countries (LMIC)

Even successful development of an effective vaccine based on a well-defined TPP can face “valleys of death” in implementation for LMIC. These result from market failures that exist for vaccines that have a low potential for return on investment and/or high development risk^117^. Various product development partnership (PDP) models, however, have been successful to develop vaccines for LMIC.

PDPs aim to share the risk and cost to accelerate vaccine development by filling market failure gaps. Some vaccines have dual high-income country and LMIC markets, such as for rotavirus and HPV, and some have primarily LMIC markets only, such as vaccines for typhoid, Japanese encephalitis, and group A meningococcal disease. None of these vaccines were developed completely de novo through the PDP model.

Significant challenges arise for PDPs when developing new vaccines for pathogens in which a vaccine currently does not exist, particularly if it is a novel pathogen and/or has a novel mechanism of action without a surrogate of protection that requires a full-fledged randomized controlled trial, such as the recently recommended malaria vaccine. These novel vaccines face inordinate investment most of which are incurred in late-stage development, creating a second valley of death that dwarfs the original one identified in early translation research and development^118^.

KSHV vaccines have the potential to overcome these barriers, but this depends on the vaccine and the target population. For example, universal childhood vaccination is mainly practical for high-risk LMIC populations, but research and development costs may in part be borne if the same vaccine is used for high-risk individuals in high-income country settings.

Six critical vaccine attributes (safety, effectiveness, product quality, supply, demand, and value) contribute to optimally achieving impact. A favorable and sustainable value proposition for a KSHV vaccine must balance affordability and acceptability against sustainability by manufacturers and those procuring the vaccine. In the past, the limited perspective on valuing vaccines just from the direct individual health benefit has left on the table the value that vaccines bring to a population, both in direct and indirect health, social, and economic benefits.

## Conclusions

Vaccine prevention or treatment of viral cancers is the underappreciated low-hanging fruit of oncology. Research on viral cancer vaccines not only has the potential to prevent a significant fraction of human cancer but may provide additional scientific benefits as well. Lessons learned from successfully developing viral cancer vaccines may provide fundamental insights into vaccines and immunotherapeutics for non-infectious cancers expressing neo-antigens.

The recent international NIH workshop on KSHV vaccines highlighted both the optimism and the barriers to reducing KSHV-related mortality and morbidity through new vaccines. A concerted effort to accurately measure the global KSHV cancer burden is needed and standardization of KSHV detection methods will be essential. The conflation of KSHV cancers with AIDS in cancer registries^119^ underestimates the burden of KSHV-related diseases, a problem that also might be exacerbated by its aggregation with unreported non-melanotic skin cancers as well as clinical diagnoses that never get recorded by registries relying on pathology-based diagnoses. Revisiting the global KSHV cancer burden and epidemiology, as has been done for other viral cancers^120,121^ is needed to guide KSHV vaccine development. The potential targets for an effective vaccine are known and new vaccine platforms for an effective vaccine hold great promise. But refocusing governmental research support for research on specific antigens and their delivery will be critical. Further, non-governmental organization support for KSHV research has been conspicuously absent yet will be critical for establishing practical and effective transnational use of KSHV vaccines. Finally, even a perfect vaccine will have no impact on KSHV-related cancer unless it is delivered to those most at risk. Since the casualties of KSHV tend to be socially and/or economically marginalized, market forces alone are unlikely to provide the resources needed for the research and development of a KSHV vaccine. Nonetheless, several important resources have been overlooked for the promotion of a practical and effective KSHV vaccine. Active HIV vaccine trial groups in highly endemic KSHV areas (e.g., Southern and Eastern Africa) may be a “two-for” since they have experience, training, and facilities from HIV vaccine clinical trials in populations that are also at high risk for KS and may be a ready platform for KSHV vaccine trials^122,123^. Making use of these existing resources would markedly reduce costs and improve clinical research for KSHV vaccine trials. Shortages of SARS-CoV-2 vaccines have also prompted the local generation of mRNA vaccines in Africa that could be repurposed to address diseases of regional importance, such as KS once the COVID-19 crisis has abated^124^. A KSHV vaccine will require a new approach to vaccine development based on the common good to ensure that a safe and effective KSHV vaccine can be delivered to those who are in the most need. Although little systematic progress has been made toward developing a KSHV vaccine, this conference highlighted paths that can be followed to achieve practical KSHV control.

### Reporting summary

Further information on research design is available in the Nature Research Reporting Summary linked to this article.

## Supplementary information

REPORTING SUMMARY
